# Novel phenotypes and genotypes in Antley-Bixler syndrome caused by cytochrome P450 oxidoreductase deficiency: based on the first cohort of Chinese children

**DOI:** 10.1186/s13023-019-1283-2

**Published:** 2019-12-30

**Authors:** Lijun Fan, Xiaoya Ren, Yanning Song, Chang Su, Junfen Fu, Chunxiu Gong

**Affiliations:** 1 Department of Endocrinology, Genetics, Metabolism, Beijing Children’s Hospital, Capital Medical University, National Center for Children’s Health, 56# Nan Lishi Rd, West District, Beijing, 100045 China; 2 Beijing Key Laboratory for Genetics of Birth Defects, Beijing Children’s Hospital, Capital Medical University, National Center for Children’s Health, 56# Nan Lishi Rd, West District, Beijing, 100045 China; 3 Department of Endocrinology, Children’s Hospital of Zhejiang University School of Medicine, Hangzhou, 310051 China

**Keywords:** Antley-Bixler syndrome, Cytochrome P450 oxidoreductase deficiency, Disorder of sex development, Congenital adrenal hyperplasia, *POR* gene

## Abstract

**Background:**

Antley-Bixler syndrome (ABS) caused by P450 oxidoreductase deficiency (PORD) is a congenital adrenal hyperplasia with skeletal malformations and disordered sex development in both sexes. There have been no reports of ABS caused by PORD in Chinese children.

**Methods:**

We described the clinical and genetic characteristics of eight Chinese children with ABS caused by PORD and compared them with those of subjects in previous studies.

**Results:**

Eight patients, aged 6 months–17.8 years, showed strikingly similar craniofacial malformations. We first described four unreported features: lower eyelid fat pads (4/8), prominent lower eyelid-zygoma transverse line (4/8), underdeveloped or absent antihelix (5/8) and single earlobe crease (5/8). Five 46, XY patients presented various degrees of undervirilization, while three 46, XX cases showed masculinization. Basal endocrine measurements revealed the following consistent results: normal cortisol; elevated adrenocorticotropic hormone, progesterone, pregnenolone, 17-hydroxypropgesterone, and corticosterone; and decreased or normal testosterone/oestradiol. We identified three previously reported variants and four novel variants (c.51719_51710delGGCCCCTGTGinsC, p.D210G, p.Y248X and p.R554X) of *POR*. The most prevalent variant was p.R457H (8/16). The hydrocortisone dosages of patients differed because of variable degrees of adrenal insufficiency.

**Conclusions:**

We described novel phenotypes and genotypes of ABS caused by PORD. The variant p.R457H was the most prevalent in this cohort. All subjects had combined characteristics of 17-hydroxylase and 21-hydroxylase deficiency. Steroid replacement therapy for patients with PORD requires individually tailored dosing.

## Background

Antley-Bixler syndrome (ABS) is a rare congenital malformation syndrome associated with midface hypoplasia, bilateral radiohumeral synostosis, multiple joint contractures, femoral bowing, long bone fractures and, occasionally, urogenital or cardiac defects [1]. ABS was first reported in 1975 [2] and was initially thought to be caused by mutations in the fibroblast growth factor receptor 2 (*FGFR2*) gene. However, in 2004, Fluck et al. [3] found that the electron donor enzyme P450 oxidoreductase (*POR*) gene mutated into another pathogenic gene of ABS combined with urogenital defects.

The *POR* gene is located at 7q11.2 and consists of 16 exons. Exons 1–15 encode for the POR protein containing 680 amino acids, while an extra first exon (1 U), which was identified later, does not encode protein [4]. POR is the electron donor for P450 enzymes, including three steroidogenic enzymes, P450c17 (17α-hydroxylase/17, 20-lyase), P450c21 (21-hydroxylase), and P450aro (aromatase), and two participating cholesterol biosynthesis enzymes, lanosterol-14α-demethylase and squalene monooxygenase [5]. POR also provides electrons to major drug-metabolizing cytochrome P450 (CYP) enzymes, among which five CYP enzymes (CYP1A2, CYP2C9, CYP2C19, CYP2D6 and CYP3A4) are responsible for the metabolism of more than 80% of all drugs [6].

P450 oxidoreductase deficiency (PORD) is a steroid synthesis disorder that is not only associated with skeletal abnormalities but also frequently presents with disorders of sex development (DSD) in affected patients [7]. Currently, approximately 100 cases of PORD have been described, and 76% of affected individuals are children [8]. However, only one case of PORD has been reported in a Chinese adult woman with “primary amenorrhea, recurrent ovarian cysts” as the main manifestations, and no cases of ABS caused by PORD in Chinese children have been reported [8]. Here, we present the first cohort of eight Chinese children with ABS caused by PORD.

## Results

### Case report

#### Patient 1

A child with a 46, XX karyotype was diagnosed with ABS caused by PORD in our centre at the age of 4.2 years. She was initially assigned as a male because of male-like external genitalia but with micropenis and unpalpable gonads. Her hearing impairment was found at 6 months and was improved by the use of a hearing aid. At the age of 2, she underwent investigations at the local hospital because of abnormal genitalia. The results revealed the 46, XX karyotype, ultrasound-confirmed dysplastic ovaries and uterus, a normal level of cortisol, elevated adrenocorticotropic hormone (ACTH) and 17-hydroxypropgesterone (17-OHP) levels, and the diagnosis of congenital adrenal hyperplasia (CAH) was made. She was treated with hydrocortisone, which was withdrawn 1 year later due to normal 17-OHP levels without any discomfort afterward. When she was referred to our centre, she was 104.5 cm in height (− 0.05 SDS), 14.5 kg in weight (− 1.13 SDS) and normotensive. We noted the following dysmorphic features: frontal bossing, lower eyelid fat pads, prominent lower eyelid-zygoma transverse line, depressed nasal bridge, underdeveloped nasal alae, pear-shaped nose, short and deep philtrum, remarkable median nodule of the upper lip, high palate, micrognathia, low-set and cupped ears, underdeveloped antihelix, single earlobe crease, cubitus valgus, bilaterally limited elbow extension, clinodactyly of the 5th fingers, and brachydactyly of distal phalanges. The phallus was 1.5 cm long with a normal male urethral meatus, fused labioscrotal folds with prominent middle ridgeline, and no palpable gonads. We suspected the diagnosis of ABS. On inquiry, the mother stated that she had developed acne and was diagnosed with polycystic ovary syndrome during pregnancy, which subsided after delivery.

#### Patient 2

Patient 2 was born at full term with a weight of 3.1 kg, and the mother developed a deep voice during pregnancy. The child presented with micropenis and hypospadias at birth and experienced urethroplasty at 1.5 years old. When he was taken to our centre at the age of 2.2 years, he was 104 cm (3.97 SDS), 17 kg (3.05 SDS) and normotensive. His distinctive appearance included frontal bossing, long eyelashes, proptosis, lower eyelid fat pads, prominent lower eyelid-zygoma transverse line, depressed nasal bridge, underdeveloped nasal alae, pear-shaped nose, short and deep philtrum, remarkable median nodule of the upper lip, high palate, micrognathia, large ears without antihelix, single earlobe crease, and clinodactyly of the 5th fingers. He presented with micropenis (stretched length, 2.6 cm), a normal urethral meatus (postoperative) and normal testes (volume, 2 ml).

#### Patient 3

Patient 3 was a 76 cm (− 0.19 SDS), 9 kg (− 1 SDS), 1-year-old boy with normal blood pressure. He was delivered by caesarean section for maternal causes at 35 weeks of gestation with a weight of 2.3 kg, micropenis and cryptorchidism. The voice of the mother had deepened during pregnancy. The child presented with dysmorphic features that included frontal bossing, lower eyelid fat pads, prominent lower eyelid-zygoma transverse line, depressed nasal bridge, underdeveloped nasal alae, pear-shaped nose, short and deep philtrum, remarkable median nodule of the upper lip, high and narrow palate, micrognathia, and asymmetrical ears (right ear was larger and lower-set) without antihelix, single earlobe crease, short neck, scoliosis, limited finger extension and micropenis (stretched length, 2.3 cm) with unilateral cryptorchidism (volume, 1 ml).

#### Patient 4

After an uneventful 39-week pregnancy without maternal virilization, a 2.90 kg boy was born to a Chinese couple. He presented with micropenis and hypospadias at birth and was first evaluated in our centre at the age of 6 months. His father had micropenis, and there was no other special family history. He was 70 cm in height (0.67 SDS), 7.6 kg in weight (− 0.9 SDS) and normotensive. His craniofacial features included lower eyelid fat pads, prominent lower eyelid-zygoma transverse line, depressed nasal bridge, underdeveloped nasal alae, pear-shaped nose, short and deep philtrum, remarkable median nodule of the upper lip, high palate and micrognathia. Examination of the external genital revealed micropenis (1.5 cm) with coronal hypospadias and testes (volume, 1 ml) in hypertrophic scrotum with a prominent middle ridgeline.

#### Patient 5

Patient 5 was a 3.5-year-old boy that was 104.9 cm in height (1.10 SDS) and 16.5 kg in weight (0.53 SDS). He was born at term with a weight of 3.6 kg. His physical characteristics included synophrys, cupped ears, high palate, low posterior hairline, single transverse palmar crease and short 3rd toes. The phallus was 2.7 cm with chordee and proximal penile hypospadias, the testes were 1–2 ml bilaterally, and the middle ridgeline was prominent.

#### Patient 6

A girl aged 17.8 years, 168 cm (1.37 SDS) and 58 kg (1.08 SDS), was referred to our hospital due to an abnormal appearance and absence of menstruation. She was born at 3.25 kg after full-term gestation with maternal masculinization as sparse hair, acne, enlargement of the nose and a deepened voice, which recovered partially after delivery. She showed congenital anomalies and sought medical treatment several times. She exhibited bilateral facial paralysis with mild cerebellar atrophy on MRI, mandibular deformity, ultrasound-confirmed infantile uterus, labial fusion, and disordered toes (the 4th toes were short and hidden in other toes); she underwent the vulvar plastic operation and excision of the 4th toes before coming to our centre. Since birth, she experienced recurrent otitis media, mild hearing impairment and spontaneous fractures of the humerus or phalanx five times. On physical examination, she presented depressed nasal bridge, underdeveloped nasal alae, pear-shaped nose, short and deep philtrum, remarkable median nodule of the upper lip, high palate, malaligned teeth, micrognathia, low-set and cupped ears without antihelix, single earlobe crease, arachnodactyly, short 4th metacarpals and thenar muscle atrophy. She showed disordered metatarsals, short 3rd toes, absence of the 4th toes (postoperative), and bilaterally limited movement of the elbows and metacarpophalangeal joints. Her breast development and pubic hair corresponded to Tanner stages IV and II, respectively.

#### Patient 7

The younger brother of patient 6 came to our centre at the age of 9.8 years. He was born by caesarean section at full term with a weight of 3 kg and a height of 50 cm. He was born with micropenis and cryptorchidism, and his mother presented with masculinization similar to that in her first pregnancy. On physical examination, he was 135.2 cm (0.69 SDS) and 32 kg (− 0.21 SDS). He showed dysmorphic features, such as pear-shaped nose with depressed nasal tip, short and deep philtrum, malaligned teeth, high palate, micrognathia, low-set and cupped ears without antihelix, concave nails, limited movement of the elbows and metacarpophalangeal joints, and short 3rd and 4th toes. The penis was 2.5 cm long, and the testes were unpalpable.

#### Patient 8

A 12.5-year-old, 143.4 cm in height (− 1.81 SDS) and 28 kg in weight (− 2.17 SDS) girl who presented with a 46, XX karyotype and normal blood pressure was evaluated for abnormal external genitalia and absence of breast development. On physical examination, she showed dysmorphic features with depressed nasal bridge, underdeveloped nasal alae, pear-shaped nose, short and deep philtrum, remarkable median nodule of the upper lip, asymmetric scapula, scoliosis, short and hidden 4th toes. Breast development and pubic hair corresponded to Tanner stage I. She presented male-like external genitalia, including clitoromegaly similar to a penis, a visible urethral opening, no vaginal opening, fused labioscrotal folds and no palpable gonads.

### Phenotypes

**Fig. 1:**
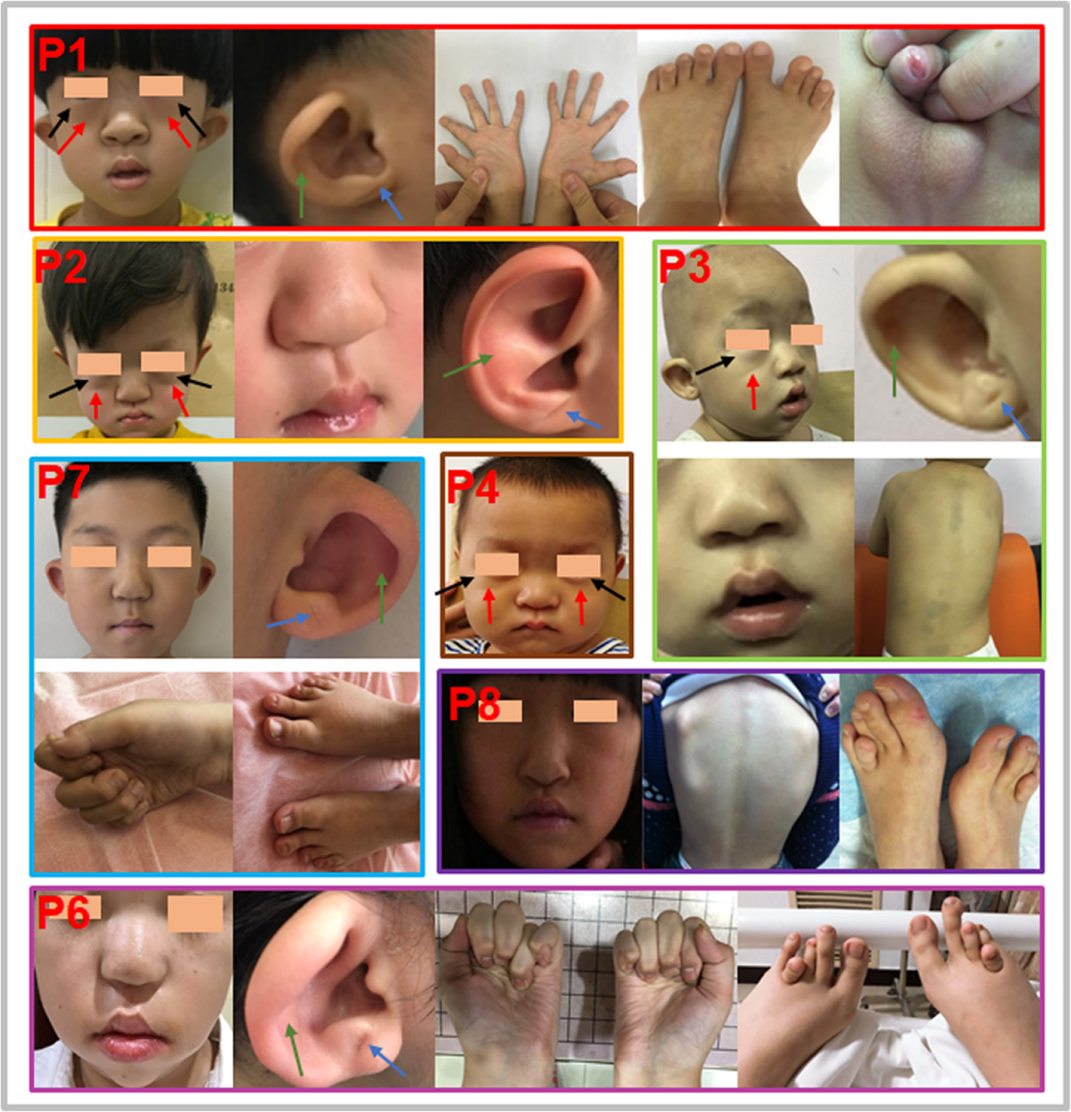
Distinctive features of Chinese children with ABS caused by PORD. Specific features are listed in Additional file 1: Table S1. The lower eyelid fat pads (black arrow), prominent lower eyelid-zygoma transverse line (red arrow), underdeveloped or absent antihelix (green arrow) and single earlobe crease (blue arrow) were first described

**Fig. 2:**
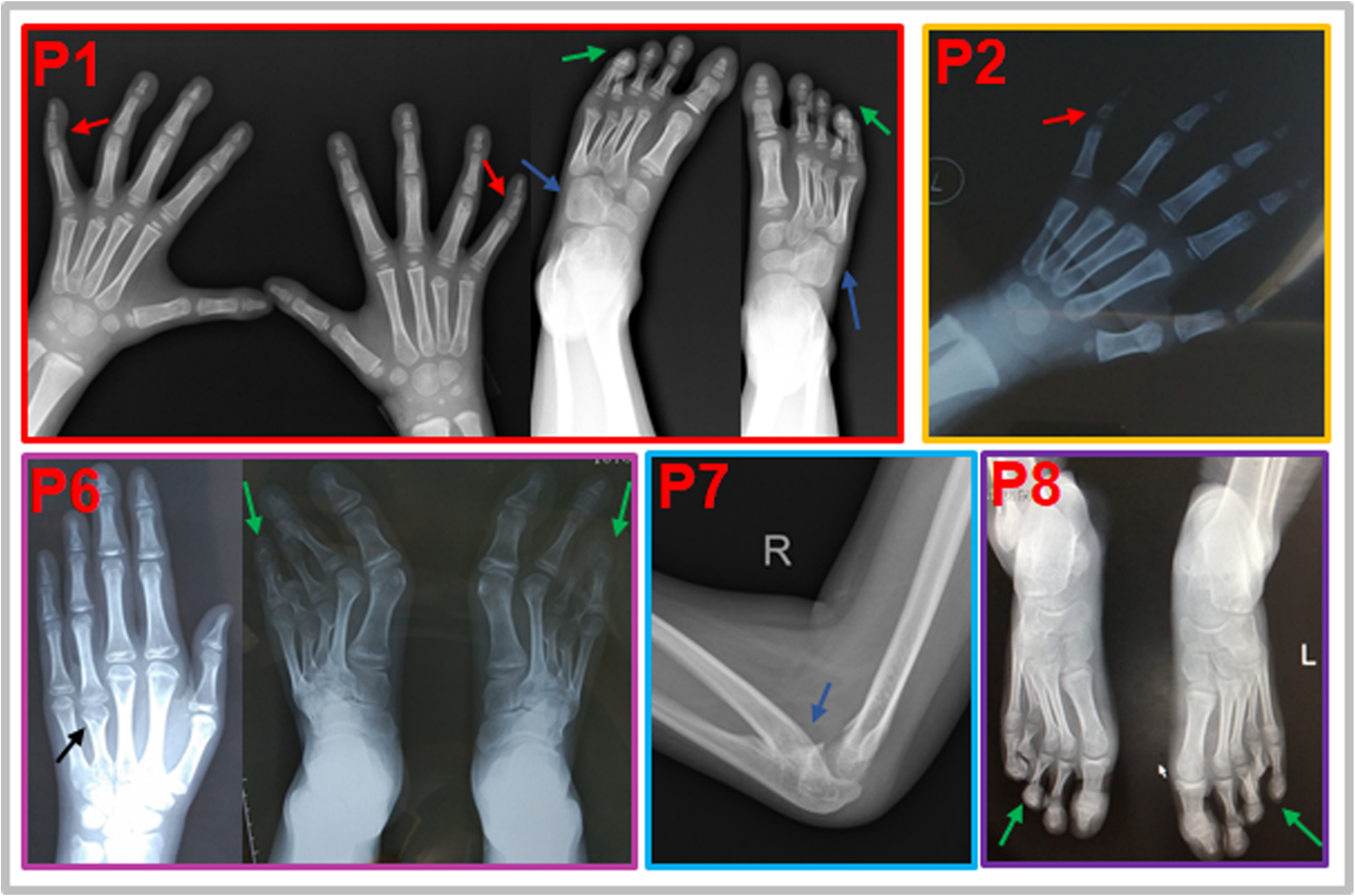
The skeletal X-ray results of Chinese children with ABS caused by PORD. Notable skeletal abnormalities included clinodactyly of the 5th fingers (red arrow), brachydactyly (green arrow, short distal phalanges for patient 1; short 3rd and 4th toes for patients 6; short 4th toes for patients 8), short 4th metacarpals (black arrow), and abnormal bone fusion (blue arrow, fusion between the lateral cuneiform bone and cuboid bone for patient 1; radioulnar synostosis for patient 7)

The special phenotypes are illustrated in Table 1, Additional file 1: Table S1, Figs. 1 and 2. All patients manifested strikingly similar facial gestalts. The lower eyelid fat pads (4/8), prominent lower eyelid-zygoma transverse line (4/8), underdeveloped or absent antihelix (5/8) and single earlobe crease (5/8) were first identified in patients with ABS. Seven individuals showed limb skeletal abnormalities, mainly presenting as limited extension of joints (4/8), brachydactyly or clinodactyly (6/8), and scoliosis (2/8). Moreover, three older subjects (patients 6, 7, 8) presented delayed bone age. All patients showed DSD in our cohort. The five 46, XY cases presented various combinations of micropenis, hypospadias and cryptorchidism. The 46, XX cases presented with masculinized genitalia: two presented with male-like external genitalia, while the other showed labial fusion, and the virilization did not progress postnatally even without treatment. Maternal virilization during pregnancy occurred in five patients and was relieved within a few weeks postpartum.

**Table 1:** Summary of the main clinical manifestations and hormonal data from the eight patients

Patient	1	2	3	4	5	6	7	8
Gender	M → F	M	M	M	M	F	M	F
Chromosome karyotype	46, XX	46, XY	46, XY	46, XY	46, XY	46, XX	46, XY	46, XX
Age at first visit	4.2 y	2.2 y	1 y	6 m	3.5 y	17.8 y	9.8 y	12.5 y
Height (cm)	104.5 (−0.05 SDS)	104 (3.97 SDS)	76 (−0.19 SDS)	70 (0.67 SDS)	104.9 (1.10 SDS)	168 (1.37 SDS)	135.2 (0.69 SDS)	143.4 (−1.81 SDS)
Weight (kg)	14.5 (−1.13 SDS)	17 (3.05SDS)	9 (−1 SDS)	7.6 (−0.9 SDS)	16.5 (0.53 SDS)	58 (1.08 SDS)	32 (−0.21 SDS)	28 (−2.17 SDS)
BA	4 y	2 y	1 y	6 m	3 y	14 y	8 y	10 y
First reported facial features								
Lower eyelid fat pads	+	+	+	+	-	-	-	-
Lower eyelid-zygoma transverse line	+	+	+	+	-	-	-	-
Underdeveloped or absent antihelix	+	+	+	-	-	+	+	-
Single earlobe crease	+	+	+	-	-	+	+	-
Skeletal abnormality								
Limited extension of joints	+	-	+	-	-	+	+	-
Brachydactyly or clinodactyly	+	+	-	-	+	+	+	+
Scoliosis	-	-	+	-	-	-	-	+
External genitalia								
Micropenis	Male	+	+	+	+	Labial	+	Male
Hypospadias	external	+	-	+	+	fusion	-	external
Cryptorchidism	genitalia	-	+	-	-		+	genitalia
Maternal prenatal virilization	+	+	+	–	–	+	+	NA
Hormone								
ACTH (pg/ml)	51.2 ↑	65.3 ↑	554 ↑	122 ↑	46.5 ↑	72.5 ↑	123 ↑	81.4 ↑
COR (ug/dl)	16.60 ↔	11.7 ↔	6.83 ↔	13.8 ↔	8.03 ↔	7.95 ↔	8.05 ↔	11.1 ↔
17-OHP (ng/ml)	12.87 ↑	11.92 ↑	8.14 ↑	6.96 ↑	5.57 ↑	4.22 ↑	7.67 ↑	NA
DHEA (ug/l)	< 0.10 ↓	< 0.10 ↓	0.18 ↔	0.3 ↔	< 0.10 ↓	1.11 ↓	0.74 ↔	NA
AD (ug/l)	< 0.10 ↓	< 0.10 ↓	0.14 ↔	< 0.10 ↓	< 0.10 ↓	0.45 ↔	< 0.10 ↓	NA
DHT (pg/ml)	25.60 ↔	158.3 ↔	112.3 ↔	476.5 ↔	< 20 ↓	47.8 ↔	< 20 ↓	NA
CORT (ug/l)	48.35 ↑	34.11 ↑	23.21↑	16.27↑	16.41↑	11.24 ↑	25.46 ↑	NA
P5 (ug/l)	5.89 ↑	4.23 ↑	6.35 ↑	5.89 ↑	5.92 ↑	6.85 ↑	13.93 ↑	NA
PGN (ng/ml)	6.19 ↑	9.96↑	23.80 ↑	16.90 ↑	3.34 ↑	5.54 ↑	12.50 ↑	NA
LH (IU/L)	0.12↔	0.25 ↔	9.30 ↑	1.02 ↔	< 0.10 ↔	25.40 ↑	0.81 ↔	9.70 ↑
FSH (IU/L)	1.44 ↔	2.75 ↔	6.59 ↑	2.49 ↔	3.45 ↑	14.8 ↑	4.99 ↔	12.7 ↑
T (ng/dl)	< 20 ↔	< 20 ↔	< 20 ↔	< 20 ↔	< 20 ↔	< 20 ↔	< 20 ↔	< 20 ↔
E2 (pg/ml)	22.1 ↔	< 20 ↔	< 20 ↔	< 20 ↔	< 20 ↔	28 ↔	< 20 ↔	< 20 ↓
Stimulated E2 or T^a^	26.80 (E2)	157 (T)	99.5 (T)	231 (T)	93 (T)	NA	NA	NA
AMH (ng/ml)	0.96	> 23 ↔	> 23 ↔	> 23 ↔	> 23 ↔	NA	NA	NA
INHB (pg/ml)	14.6	17.9 ↓	215 ↔	279.31 ↔	96 ↔	NA	NA	NA
Ultrasound								
Adrenal gland	normal	normal	normal	normal	normal	normal	normal	hyperplasia
Gonads	dysplastic ovaries infant uterus	testes	testes	testes	testes	ovarian cysts infant uterus	testes	dysplastic ovaries infant uterus
Treatment H-C (mg/m2/day)	16.46	6.63	12.05	27.32	29.52	12.42	18.35	18.52
*POR* gene variants	c.1370G > A, p.R457H (P)	c.1370G > A, p.R457H (P)	c.1370G > A, p.R457H (P)	c.1370G > T, p.R457L (P)	c.1370G > A, p.R457H (P)	c.1370G > A, p.R457H (P)	c.1370G > A, p.R457H (P)	c.1370G > A, p.R457H (P)
	c.744C > G, p.Y248X^b^ (P)	c.744C > G, p.Y248X^b^ (P)	c.1660C > T, p.R554X^b^ (P)	c.1820A > G, p.Y607C (LP)	c.629A > G, p.D210G^b^ (LP)	c.517-19_517-10delGGCCCCTGTGinsC^b^ (P)	c.517-19_517-10delGGCCCCTGTGinsC^b^ (P)	c.1370G > A, p.R457H (P)

Notes: Patients 1 to 7 were from Beijing Children’s Hospital, and patient 8 was from Children’s Hospital of Zhejiang University School of Medicine

*M* male, *F* female, *M → F* male switch to female, *y* year, *m* month, *SDS* standard deviation score, *BA* bone age, *NA* data not available, *H-C* hydrocortisone, *P* Pathogenic, *LP* likely pathogenic, *ACTH* adrenocorticotropic hormone, *COR* cortisol, *17-OHP* 17-hydroxypropgesterone, *DHEA* dehydroepiandrosterone, *AD* androstenedione, *DHT* dihydrotestosterone, *CORT* corticosterone, *P5* pregnenolone, *PGN* progesterone, LH luteinizing hormone, *FSH* follicle-stimulating hormone, *T* testosterone, *E2* oestradiol, *AMH* anti-Mullerian hormone, *INHB* inhibin B

+, finding present; −, finding absent; ↑ represents the value was above the normal range; ↔ represents the value was within the normal range; ↓ represents the value was below the normal range

^a^T and E2 were detected after human chorionic gonadotropin (hCG) stimulation test and human menopausal gonadotropin (hMG) stimulation test, respectively. ^b^Represents the first reported variants

### Laboratory findings

Patients in the cohort exhibited consistent hormone profiles (Table 1). All patients showed normal cortisol but elevated ACTH levels. The ACTH stimulation test (250 μg) in patient 1 showed a poor response (basal cortisol of 14.6 μg/dl with a subnormal response to 13.7 μg/dl and basal 17-OHP of 12.9 ng/ml with a poor response to 24.8 ng/ml after 60 min). Ultrasound revealed normal adrenal glands except for mild adrenal hyperplasia in patient 8. Except for patient 8 without corresponding hormone results, the remaining seven patients all showed decreased or normal baseline dehydroepiandrosterone (DHEA), androstenedione (AD) and dihydrotestosterone (DHT) levels, while serum progesterone (PGN), pregnenolone (P5), 17-OHP and corticosterone (CORT) levels were elevated. For the 46, XY patients, basal levels of testosterone (T) were undetectable, and gonadotropins were elevated in patients 3 and 5. The basal concentrations of anti-Mullerian hormone (AMH) and inhibin B (INHB) were within the normal range in patients 3, 4, and 5, while INHB was decreased in patient 2. However, stimulated T maintained a normal level in four males who were subjected to human chorionic gonadotropin (hCG) stimulation test. Three 46, XX patients also showed signs of gonadal dysplasia: oestradiol (E2) responded poorly to human menopausal gonadotropin (hMG) stimulation test in patient 1; gonadotropins were elevated but E2 was normal in patient 6 and decreased in patient 8; ultrasound revealed an infantile uterus in all 46, XX patients, dysplastic ovaries in patients 1 and 8 and ovarian cysts in patient 6.

### Mutational analysis

As shown in Table 1, we detected seven different *POR* variants, with p.R457H being the most prevalent (8/16). Seven patients were compound heterozygous for p.R457H or p.R457L with other variants (6 with p.R457H, 1 with p.R457L), while one was homozygous for p.R457H. The p.D210G, p.Y248X, p.R554X and c.51719_51710delGGCCCCTGTGinsC variants are being reported for the first time. All variants were evaluated as being pathogenic or likely pathogenic according to American College of Medical Genetics and Genomics and the Association for Molecular Pathology (ACMG/AMP) guidelines [9]. Furthermore, the three-dimensional (3D) protein models of *POR* revealed that the novel missense variant p.D210G destroys hydrogen bonds, which may lead to instability of the protein structure (Additional file 2: Figure S1).

### Treatment

After diagnosis, all subjects received hydrocortisone treatment (the dosages are shown in Table 1). Patient 1 was reassigned to the female gender. Patients 1 and 8 subsequently underwent vulvar plastic surgery. Five male patients were treated with oral testosterone to treat micropenis, and patients 6 and 8 began menstruating 2 months and 6 months after receiving oestradiol replacement therapy, respectively.

## Discussion

ABS caused by PORD presented with variable phenotypes. We described similar craniofacial malformations and reported four novel features in the first Chinese cohort. Remarkably, affected subjects have also been reported to have otologic abnormalities such as stenotic external auditory canals and middle or inner ear malformations, which may lead to recurrent otitis media and conductive hearing loss [10, 11]. This finding can also explain the hearing impairment in patients 1 and 6. Interestingly, we also observed facial paralysis in patient 6, which may be attributed to an abnormal course or compression of the facial nerve caused by severe craniofacial abnormalities [11]. Moreover, seven patients in the present study showed limb skeletal malformations, and most were mild. ABS is difficult to identify because certain skeletal malformations may gradually appear with age, and facial abnormalities are the most recognizable figures, especially in children.

Decreased activity of 17, 20-lyase and aromatase lead to disruption of both androgen and oestrogen synthesis, which contributes to DSD in both sexes [7]. Male patients in the cohort presented with undervirilization, while the mothers and female patients showed prenatal masculinization. The prenatal androgen excess was related to impairment of placental aromatase and/or activation of the “backdoor pathway” of androgen biosynthesis, which closes shortly after birth [12]. However, there is a striking contradiction, i.e., clinical evidence of antenatal maternal masculinization but prenatal androgen deficiency in affected males, that has not been mentioned to date. In fact, maternal masculinization occurs in the second trimester of pregnancy [12], while male external genitalia are entirely formed at the 12-week embryo stage [13].

There was evidence of gonadal dysplasia in our cohort. The elevated basal gonadotropins in patients 3, 5, 6 and 8 indicated hypergonadotropic hypogonadism. Low levels of INHB in patient 2 manifested as Sertoli cell dysplasia. The poor response to hMG stimulation in patient 1, ultrasound-confirmed infantile uteruses in female patients and ovarian cysts in patient 6 also indicated dysplastic ovaries. Most reported subjects showed delayed puberty, but spontaneous progression during puberty has also been observed [14]. Additionally, ovarian cysts have been reported in a number of adolescent and adult females with PORD [14, 15], which may be related to high gonadotropin-induced ovarian hyperplasia caused by decreased oestrogen. We should pay attention to pubertal development during follow-up.

Subjects with biallelic *POR* variants show the combined enzymatic deficiency of CYP21A2 and CYP17A1 [16]. Patient 1 showed a normal basal cortisol level but adrenal insufficiency after ACTH stimulation test, indicating that ACTH stimulation test is mandatory for assessing adrenal function. Although the basal ACTH levels of patients 3, 4, and 7 were elevated significantly, none of them showed significant clinical signs of adrenal insufficiency, suggesting that adrenal dysfunction caused by PORD is difficult to notice if facial and skeletal abnormalities are not remarkable. Therefore, affected subjects are at a high risk for developing a life-threatening adrenal crisis under stress. In addition, mineralocorticoid excess due to inhibition of 17α-hydroxylase activity can result in hypertension, which typically manifests in young adulthood [10]. While all patients in this group were normotensive upon examination, more attention should be paid during follow-up.

Since *POR* was identified as the pathogenic gene of ABS, more than 90 variants have been identified (http://www.hgmd.cf.ac.uk/ac/index.php). Different variants have been described in specific ethnic groups, such as p.A287P among Europeans [17] and p.R457H among Japanese individuals [18]. The high prevalence of p.R457H in the Japanese population and our cohort is likely attributed to a founder effect in Asians.

The diversity of *POR* gene variants may contribute to the variable phenotypes and hormone profiles of PORD. Research has shown that patients with nonsense mutations present more severe phenotypes than those with missense mutations [17]. Similarly, in our cohort, subjects (patients 1, 2, 3, 6, 7) carrying the nonsense mutation or splicing mutation showed more severe craniofacial and skeletal abnormalities. Different variants may cause variable enzymatic deficiency, and the phenotypes of patients vary considerably. The p.R457H variant abolished all measurable activity of CYP19A1, similar to CYP17A1 activities [19]. In contrast, p.A287P was found to decrease 17α-hydroxylase to 20% and 17, 20-lyase to 10% of wild-type activity but exerted no inhibitory effect on CYP19A1 activity [3, 19]. Consistent with this finding, patients with p.R457H tend to be associated with severe skeletal phenotypes and maternal virilization during pregnancy, while patients with p.A287P are less severely affected [20]. Consistently, five of seven patients carrying p.R457H showed maternal masculinization during pregnancy. Further studies are required to elucidate the correlation between genotype and phenotype.

The ACTH stimulation test should be performed to evaluate the adrenal function of ABS patients with PORD, and if indicated, glucocorticoid replacement is appropriate. Twenty-four of 27 patients with PORD were found to have adrenal insufficiency after ACTH stimulation, 13 received permanent hydrocortisone replacement, and the remaining 11 required hydrocortisone treatment only during stress [17]. Our patients showed variable degrees of adrenal insufficiency, and the dosages of hydrocortisone differed. Patients with PORD usually require lower doses of hydrocortisone than those with classic CAH [7, 15], as PORD leads to partial deficiency of steroidogenic enzymes, serum ACTH and 17-OHP levels are lower, and there is no postnatal androgen excess. Additionally, decreased activity of the liver P450 enzyme impairs hepatic drug and steroid metabolism in patients with PORD. Therefore, steroid replacement therapy, including glucocorticoid and sex steroids, requires individually tailored dosing [6].

## Conclusion

ABS caused by PORD is a rare disease that is easily misdiagnosed if adrenal insufficiency is not remarkable. The distinctive facial appearance, skeletal abnormalities, DSD, adrenal insufficiency and maternal antenatal masculinization suggest the diagnosis. This study presented the first cohort of Chinese children with ABS caused by PORD. We also described four unreported craniofacial malformations and four novel *POR* variants, enriching the phenotypic and genotypic spectrum. The p.R457H variant, which was previously reported to be common in Japanese individuals, was also the most prevalent mutation in this Chinese cohort, suggesting that p.R457H is common in Asians. Steroid replacement therapy for patients with PORD requires individually tailored dosing.

## Patients and methods

### Patients

Patients 1 to 7 were from Beijing Children’s Hospital, and patient 8 was from Children’s Hospital of Zhejiang University School of Medicine.

### Mutational analysis

All patients were subjected to targeted sequencing with a gene panel consisting of 127 DSD- and adrenal-related genes or whole-exome sequencing (WES) for next-generation sequencing (NGS) in family trios. The candidate variants were confirmed by Sanger sequencing. The reference sequence of *POR* was NM_000941.2, and the pathogenic evaluation of variants was based on ACMG/AMP guidelines [9].

## Supplementary information

**Additional file 1: Table S1.** Specific features of eight Chinese children with ABS.

**Additional file 2: Figure S1.** The 3D protein models of mutant POR (p.D210G). The 3D protein models of POR were based on the crystal structure (Protein Data Bank code 6 J79) using PyMOL (Version 1.3, Schro-dinger, LLC). Wild-type p.D210 (a) and the mutated p.D210G (b) forms are depicted in pink and yellow, respectively. The residue p.D210 is between the β-strands and the loops, forming two hydrogen bonds with the p.K179 and p.D212 residues. The p.D210G mutation disrupts those two hydrogen bonds and forms a new hydrogen bond with a loop of the G213 residue

## Data Availability

Data are available in a public, open access repository. All data relevant to the study are included in the article or uploaded as supplementary information.

## Abbreviations

17-OHP: 17-hydroxypropgesterone
3D: Three-dimensional
ABS: Antley-Bixler syndrome
ACMG/AMP: American College of Medical Genetics and Genomics/Association for Molecular Pathology
ACTH: Adrenocorticotropic hormone
AD: Androstenedione
AMH: Anti-Mullerian hormone
CAH: Congenital adrenal hyperplasia
COR: Cortisol
CORT: Corticosterone
DHEA: Dehydroepiandrosterone
DHT: Dihydrotestosterone
DSD: Disorders of sex development
E2: Oestradiol
*FGFR2*: Fibroblast growth factor receptor 2
FSH: Follicle-stimulating hormone
hCG: Human chorionic gonadotropin
hMG: Human menopausal gonadotropin
INHB: Inhibin B
LH: Luteinizing hormone
LP: Likely pathogenic
NGS: Next-generation sequencing
P: Pathogenic
P5: Pregnenolone
PGN: Progesterone
*POR*: Electron donor enzyme P450 oxidoreductase
SDS: Standard deviation score
T: Testosterone
WES: Whole-exome sequencing
