# Supplementary material for: Novel phenotypes and genotypes in Antley-Bixler syndrome caused by cytochrome P450 oxidoreductase deficiency: based on the first cohort of Chinese children
Source: Orphanet J Rare Dis. 2019 Dec 30;14:299. doi: 10.1186/s13023-019-1283-2 (PMC6937861; doi:10.1186/s13023-019-1283-2)
Supplement: Supplementary file 1 — Additional file 1: Table S1. Specific features of eight Chinese children with ABS. [file 13023_2019_1283_MOESM1_ESM.docx]

**Table S1 Specific features of eight Chinese children with ABS.**

| **Patient** | **1** | **2** | **3** | **4** | **5** | **6** | **7** | **8** |
| --- | --- | --- | --- | --- | --- | --- | --- | --- |
| **Head**  Frontal bossing | + | + | + | - | - | - | - | - |
| **Eyes**  Proptosis  Lower eyelid fat pads  Lower eyelid-zygoma transverse line | -  +  + | +  +  + | -  +  + | -  +  + | -  -  - | -  -  - | -  -  - | -  -  - |
| **Nose**  Depressed nasal bridge  Underdeveloped nasal alae  Pear-shaped nose | +  +  + | +  +  + | +  +  + | +  +  + | -  -  - | +  +  + | -  +  + | +  +  + |
| **Mouth**  Short and deep philtrum  Remarkable median nodule of the upper lip  High palate  Micrognathia | +  +  +  + | +  +  +  + | +  +  +  + | +  +  +  + | -  -  +  - | +  +  +  + | +  -  +  + | +  +  -  - |
| **Ears**  Low-set ears  Cupped ears  Underdeveloped or absent antihelix  Single earlobe crease | +  +  +  + | -  -  +  + | +  +  +  + | -  -  -  - | -  +  -  - | +  +  +  + | +  +  +  **+** | NA  NA  NA  NA |
| **Skeletal abnormalities**  Scoliosis  Cubitus valgus  Brachydactyly  Clinodactyly of the 5th fingers  Limited extension of joints | -  +  + (distal phalanges)  +  + (elbows) | -  -  -  +  - | +  -  -  -  + (fingers) | -  -  -  -  - | -  -  + (3rd toes)  -  - | -  -  + (3rd, 4th toes)  -  + (elbows, fingers) | -  -  + (3rd, 4th toes)  -  + (elbows, fingers) | +  -  + (4th toes)  -  - |

+, finding present; −, finding absent; NA, data not available.
