# Supplementary figures and images for: Novel phenotypes and genotypes in Antley-Bixler syndrome caused by cytochrome P450 oxidoreductase deficiency: based on the first cohort of Chinese children
Source: Orphanet J Rare Dis. 2019 Dec 30;14:299. doi: 10.1186/s13023-019-1283-2 (PMC6937861; doi:10.1186/s13023-019-1283-2)

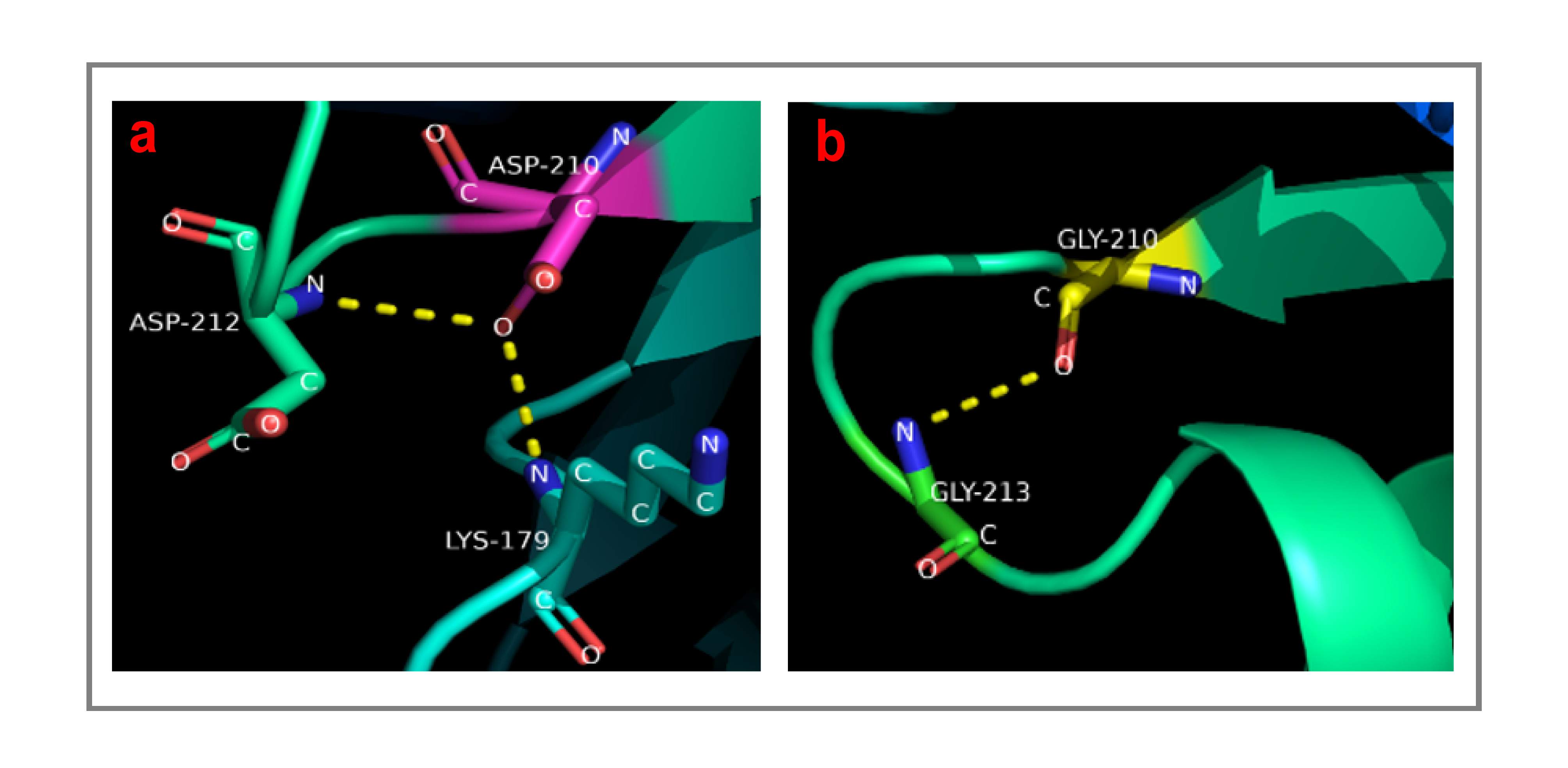

Supplement: Supplementary file 2 — Additional file 2: Figure S1. The 3D protein models of mutant POR (p.D210G). The 3D protein models of POR were based on the crystal structure (Protein Data Bank code 6 J79) using PyMOL (Version 1.3, Schro-dinger, LLC). Wild-type p.D210 (a) and the mutated p.D210G (b) forms are depicted in pink and yellow, respectively. The residue p.D210 is between the β-strands and the loops, forming two hydrogen bonds with the p.K179 and p.D212 residues. The p.D210G mutation disrupts those two hydrogen bonds and forms a new hydrogen bond with a loop of the G213 residue [file 13023_2019_1283_MOESM2_ESM.tif]
